# Tau Fibril Formation in Cultured Cells Compatible with a Mouse Model of Tauopathy

**DOI:** 10.3390/ijms19051497

**Published:** 2018-05-17

**Authors:** Gen Matsumoto, Kazuki Matsumoto, Taeko Kimura, Tetsuya Suhara, Makoto Higuchi, Naruhiko Sahara, Nozomu Mori

**Affiliations:** 1 Department of Anatomy and Neurobiology, Nagasaki University School of Medicine, 1-12-4 Sakamoto, Nagasaki 852-8523, Japan; bb20113096@ms.nagasaki-u.ac.jp (K.M.); morinosm@nagasaki-u.ac.jp (N.M.); 2 Department of Functional Brain Imaging Research, National Institute of Radiological Sciences, National Institutes for Quantum and Radiological Science and technology, 4-9-1 Anagawa, Inage, Chiba 263-8555, Japan; kimura.taeko@qst.go.jp (T.K.); suhara.tetsuya@qst.go.jp (T.S.); higuchi.makoto@qst.go.jp (M.H.)

**Keywords:** tauopathy, cellular model, sarkosyl insoluble tau, super-resolution microscopy

## Abstract

Neurofibrillary tangles composed of hyperphosphorylated tau protein are primarily neuropathological features of a number of neurodegenerative diseases collectively termed tauopathy. To understand the mechanisms underlying the cause of tauopathy, precise cellular and animal models are required. Recent data suggest that the transient introduction of exogenous tau can accelerate the development of tauopathy in the brains of non-transgenic and transgenic mice expressing wild-type human tau. However, the transmission mechanism leading to tauopathy is not fully understood. In this study, we developed cultured-cell models of tauopathy representing a human tauopathy. Neuro2a (N2a) cells containing propagative tau filaments were generated by introducing purified tau fibrils. These cell lines expressed full-length (2N4R) human tau and the green fluorescent protein (GFP)-fused repeat domain of tau with P301L mutation. Immunocytochemistry and super-resolution microscopic imaging revealed that tau inclusions exhibited filamentous morphology and were composed of both full-length and repeat domain fragment tau. Live-cell imaging analysis revealed that filamentous tau inclusions are transmitted to daughter cells, resulting in yeast-prion-like propagation. By a standard method of tau preparation, both full-length tau and repeat domain fragments were recovered in sarkosyl insoluble fraction. Hyperphosphorylation of full-length tau was confirmed by the immunoreactivity of phospho-Tau antibodies and mobility shifts by sodium dodecyl sulfate-polyacrylamide gel electrophoresis (SDS-PAGE). These properties were similar to the biochemical features of P301L mutated human tau in a mouse model of tauopathy. In addition, filamentous tau aggregates in cells barely co-localized with ubiquitins, suggesting that most tau aggregates were excluded from protein degradation systems, and thus propagated to daughter cells. The present cellular model of tauopathy will provide an advantage for dissecting the mechanisms of tau aggregation and degradation and be a powerful tool for drug screening to prevent tauopathy.

## 1. Introduction

The microtubule-associated protein tau abnormally aggregates into intracellular, filamentous inclusions (neurofibrillary tangles; NFTs) in the brains of individuals with neurodegenerative disorders termed tauopathies [1]. The primary function of tau protein, which is normally localized in the axons of neurons, is to stabilize microtubules. With its ability to modulate microtubule dynamics, tau contributes to key structural and regulatory cellular functions, such as maintaining neuronal processes and regulating axonal transport, respectively. The normal dynamic equilibrium of microtubule-bound tau is primarily determined by the phosphorylation state of tau. Microtubule-bound tau is promoted by the dephosphorylation of tau, and detachment of tau from microtubules is promoted by their phosphorylation of tau. Filamentous tau in tauopathy brains is abnormally hyperphosphorylated [2]. However, it remains unresolved whether tau phosphorylation is the causative for filamentous tau aggregation. One could argue that recombinant tau without phosphorylation is able to self-assemble into filaments [3,4]. Other post-translational modifications may contribute to tau self-assembly. Nevertheless, tau filament formation is one of the major processes toward the onset of neurodegenerative disease.

Cellular and animal models of tauopathy are essential for the development of diagnostic and therapeutic procedures. Until recently, the overproduction of wild-type tau in stable cell lines has not led to robust tau aggregations [5,6,7,8] except a cell line with conditional expression [9]. Instead of overexpression models, introducing tau seeds into tau-expressing cells successfully generated tau aggregation [10,11,12,13,14]. These tau seeds were derived from brain extracts of tauopathy patients or tau transgenic mice, cell lysates from tau-aggregate bearing cells, or recombinant tau fibrils. Conditioned media from cells with tau aggregates were also able to induce aggregation in recipient cells [15,16]. However, to our knowledge, stable cell lines with filamentous tau aggregates are not widely distributed in the research field. On the other hand, the discovery of the microtubule-associated protein tau (MAPT) mutations in familial frontotemporal lobar degeneration with underlying tau pathology (FTLD-Tau) facilitated the development of tau transgenic mouse models mimicking salient features of diseases. Previously, Lewis and Ashe generated an inducible mouse model expressing human 0N4R tau with the P301L mutation, termed rTg4510 mice [17]. Expression of human tau is controlled by the tetracycline transactivator transgene under the calcium/calmodulin-dependent protein kinase IIα (CaMKIIα) promoter. This mouse line develops progressive intracellular tau aggregations in corticolimbic areas and forebrain atrophy. Biochemical examinations revealed that neurofibrillary tangles (NFTs) in this model are accompanied by sarkosyl-insoluble, hyperphosphorylated tau that migrated at 64 kDa [18]. Although animal models have an advantage for preclinical studies, relevant cellular models are still useful for the first screening of a drug efficacy.

In this study, we aimed to generate a novel cellular model of filamentous tau aggregate formation. We developed stable cell lines, in which full-length human tau and the green fluorescent protein (GFP)-fused repeat domain (K18) of tau with the P301L mutation were co-expressed, forming propagative aggregates. The generated cell lines were examined for morphological and biochemical properties of tau inclusions, and were then compared with these properties in the rTg4510 tauopathy mouse model.

## 2. Results

### 2.1. Generation of Stable Cell Lines with Intracellular Tau Aggregates

**Figure 1:**
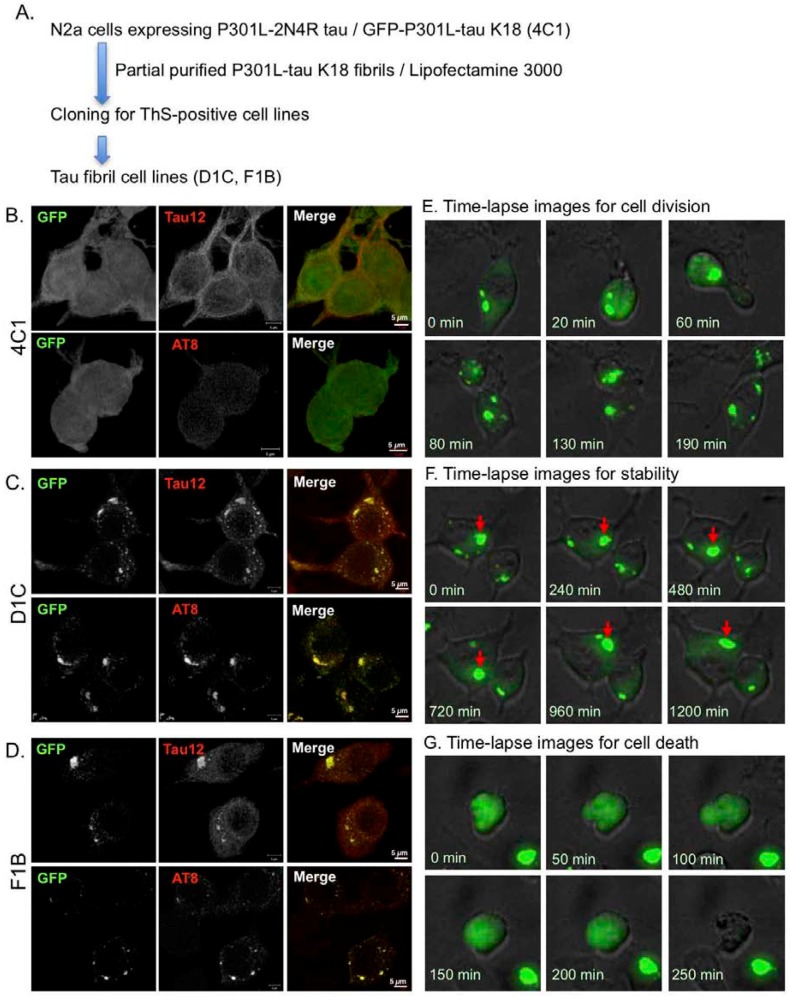
Stable Neuro2a cell lines expressing the human 2N4R tau isoform and repeat domain fragment with the P301L mutation. (**A**) Schematic representation of cloning for tau fibril cell lines. To generate cells with Tau inclusions, recombinant K18 tau fibrils were transduced into 4C1 cells by lipofectamine 3000. Single cells with GFP-positive inclusion were cloned for generating stable cell lines; (**B**–**D**) GFP fluorescence imaging and immunocytochemistry of stable Neuro2a cell lines ((**B**), 4C1 cell; (**C**), D1C cell; (**D**), F1B cell). Methanol-formaldehyde fixed cells were immunolabeled with the Tau12 or AT8 antibody. GFP signal and immunoreactivity were captured by fluorescence microscopy with green and red filters, respectively. Co-localization with GFP and immunoreactivity was indicated by a yellow signal in merged images; (**E**–**G**) Time-lapse images of the F1C cell during cell division (**E**), stable period (**F**), and cell death (**G**). GFP fluorescence was captured by an incubator microscope. GFP inclusions were transmitted from mother to daughter cells within an hour (**E**). Representative images showed a long living inclusion of more than 20 h ((**F**); red arrow indicates a stable inclusion). The other representative images show a bursting GFP inclusion during cell death (**G**).

To develop cellular models for tau aggregation, we first generated a Neuro2a (N2a) cell line (4C1) stably expressing both the full-length 2N4R tau isoform and GFP-K18 (green fluorescent protein (GFP)-fused repeat domain of tau, Q244-E372) with the P301L FTDP-17-tau mutation. The fluorescence signals of GFP-K18 were diffusively detected throughout the cells and Tau12 antibody immuno-labeled full-length Tau proteins were observed on microtubules by confocal fluorescence microscopy, while AT8 immunofluorescence was rarely detectable (Figure 1B). To generate intracellular tau aggregates, based on previous reports, purified K18 tau fibrils were transduced into 4C1 cells by lipofectamine [19,20]. After incubation for 10–14 days, single colonies with condensed GFP fluorescence signals were picked up, and after three or more sub-cloning processes, clonal cultures were established. Clones D1C and F1B had large numbers of GFP-positive inclusions that were co-labeled with both Tau12 and AT8 antibodies, suggesting that both full-length and K18 tau were incorporated in them and the full-length Tau was phosphorylated (Figure 1C,D). Time-lapse imaging revealed that tau aggregates grew larger for varying intervals and transmitted to daughter cells during the cell division (Figure 1E). Many of the inclusions were stable in cytosolic compartments for more than 20 h (Figure 1F). Round-shaped cells with large-size inclusions with intense GFP signals were occasionally observed and they eventually exhibited cell death (Figure 1G). These results indicate that the Tau aggregates propagate in a yeast-prion-like manner, that is, a transmission of prions to daughter cells during cell division [21].

### 2.2. Tau Biochemistry in Tau Fibril Cell Lines

**Figure 2:**
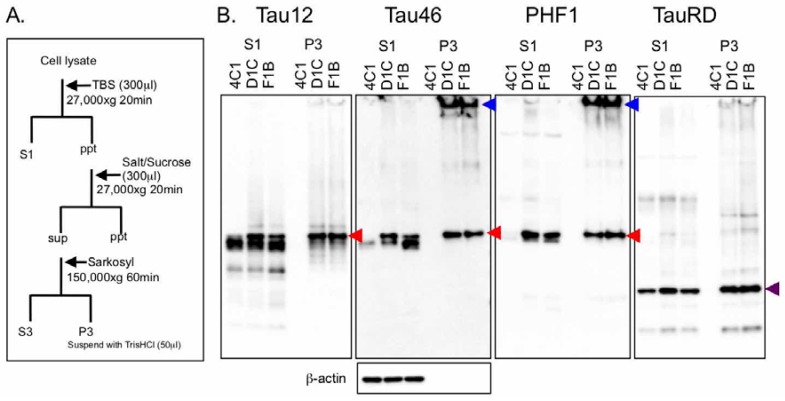

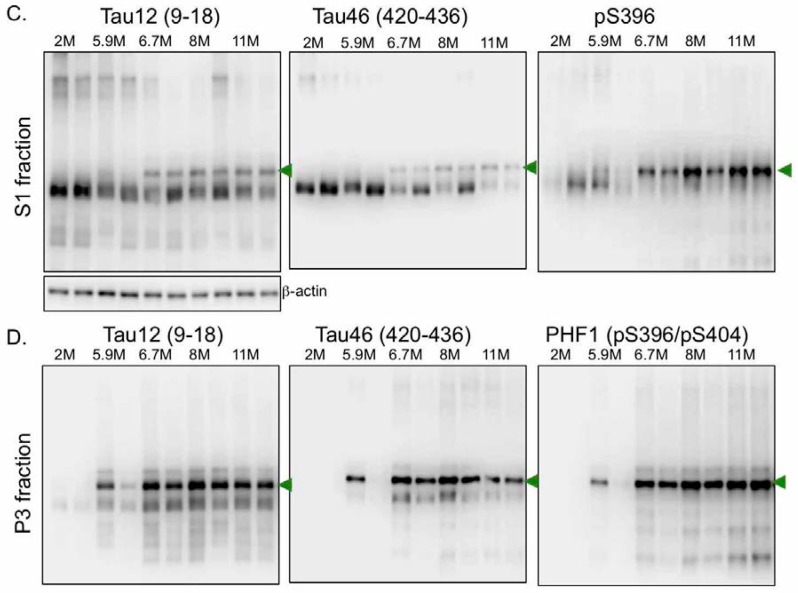
Biochemical characterization of tau protein in cells and mouse brains. (**A**) Cellular fractionation protocol for detecting tau protein in TBS-extractable (S1) and sarkosyl-insoluble fractions (P3) from 4C1, D1C, and F1B cells; (**B**) 2.5 μL of each sample from fractionated cell lysate was loaded on gel and SDS-PAGE was performed. Subsequently, western blots of S1 and P3 fractions with Tau12, Tau46, PHF1, TauRD, and β-actin antibodies were carried out. All cell lines (4C1, D1C, and F1B) expressed both human full-length tau (65–72 kDa) and GFP-K18 (43 kDa, purple arrowhead) recognized by Tau12 and TauRD antibodies, respectively. Both full-length and GFP-K18 tau were recovered in P3 fractions of D1C and F1B cells, but not in the P3 fraction of the 4C1 cell. High molecular weight aggregates (blue arrowheads) also appeared in stacking gel of P3 fractions of D1c and F1B cells. Hyperphosphorylated tau (72 kDa, red arrowheads) was detected in S1 and P3 fractions of D1C and F1B cells; (**C**) Western blots for detecting TBS-extractable tau in rTg4510 mice. A certain amount of S1 fraction (loading sample containing 0.01 mg wet-weight of brain) from 2-, 5.9-, 6.7-, 8-, and 11-month-old rTg4510 mice was separated by SDS-PAGE, and then western blotting with Tau12, Tau46, pS396, PHF1, and β-actin antibodies was conducted. Green arrowhead indicates hyperphosphorylated 64 kDa tau. Mobility shift of full-length 0N4R tau (50–60 kDa to 64 kDa) was clearly observed from 5.9- to 6.7-month-old rTg4510 mice; (**D**) Western blots for detecting sarkosyl-insoluble tau in rTg4510 mice. A certain amount of P3 fraction (loading sample containing 0.5 mg wet-weight of brain) from the above-mentioned mice was separated by SDS-PAGE, and then western blotting with E1, Tau46, and PHF1 antibodies was conducted. Green arrowhead indicated hyperphosphorylated 64 kDa tau.

There is a standard biochemical protocol to isolate tau aggregates, as reported by Greenberg and Davies [22]. Filamentous tau was enriched in the sarkosyl-insoluble fraction from human Alzheimer’s brain extracts, and it was characterized by its paired helical filament (PHF) structure. In this study, we used a conventional method for isolating filamentous tau aggregates [23] from the aforementioned stable cell lines. As observed by fluorescence images, the 4C1 cell contained a buffer-extractable full-length 2N4R tau isoform and K18 tau with less phosphorylation, but immunoreactivities of tau antibodies were not observed in the sarkosyl-insoluble fraction (P3 fraction) (Figure 2B). On the other hand, both D1C and F1B cells showed PHF1-positive full-length tau with a smear pattern in the P3 fraction (Figure 2B). Robust immunoreactivities of Tau46 and PHF1 antibodies appeared in stacking gel space, suggesting the existence of highly aggregated tau proteins (Figure 2B). K18 tau from D1C and F1B cells was recovered more in the P3 fraction than the S1 fraction from D1C and F1B cells (Figure 2B). A difference between D1C and F1B cells was observed in the S1 fraction, which is a more phosphorylated 72 kDa band in the D1C cell than in the F1B cell (Figure 2B). Correspondingly, hyperphosphorylated tau bands migrating to 64 kDa in both S1 and P3 fractions were significant features of the rTg4510 tauopathy mouse model expressing the 0N4R tau isoform with the P301L mutation (Figure 2C,D). These tau bands had both amino- and carboxyl-terminals of tau epitopes. The 64 kDa band in the S1 fraction appeared from 6.7 months of age, while that in the P3 fraction was detected in 5.9-month-old mice and then increased with age. Since these hyperphosphorylated tau bands were strongly associated with tau pathology in rTg4510 mice, the 72 kDa band corresponding to the hyperphosphorylated 2N4R tau isoform observed in D1C and F1B cells was most likely a pathogenic form of the tau protein.

### 2.3. Super-Resolution Microscopic Analysis for Detecting Filamentous Tau Aggregates

**Figure 3:**
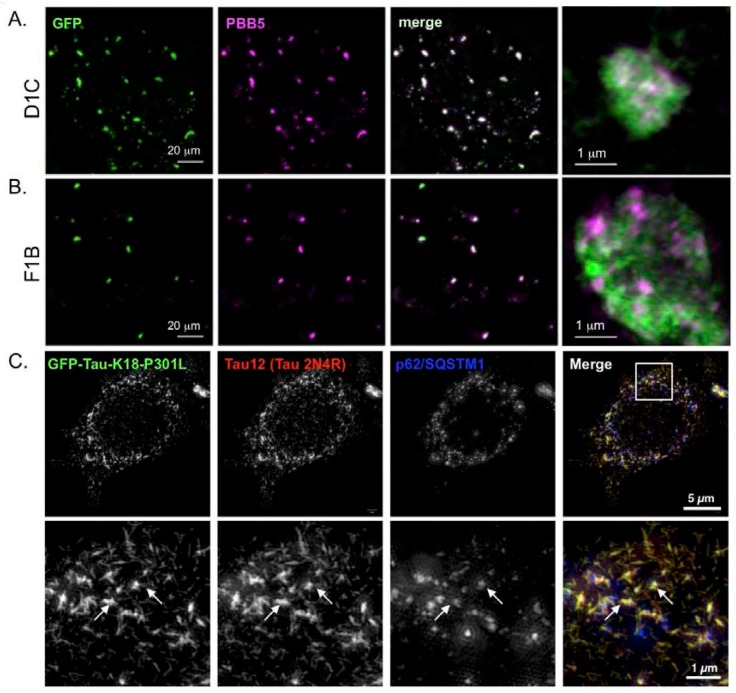

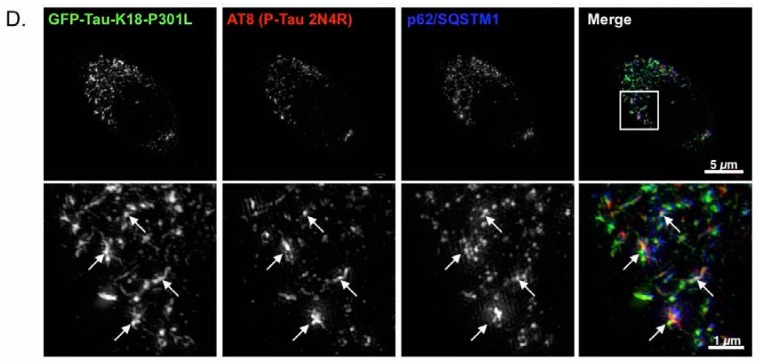
Visualization of cellular tau aggregates in tau fibril cell lines. (**A**,**B**) D1C (**A**) and F1B (**B**) cells labeled by the PBB5 ligand were captured with GFP and Cy3 filters to detect GFP and PBB5 fluorescence, respectively. Both cell lines showed co-localization of GFP and PBB5 signals, indicating the existence of tau fibrils composed of β-sheets in both cell lines. Bars = 20 μm. Right end panels showed high-magnified images detecting GFP (green) and PBB5 (purple) signals. Bars = 1 μm; (**C**) F1B cell was immunostained with Tau12 and p62 antibodies following formaldehyde fixation, and visualized by super-resolution structured illumination microscopy (SR-SIM). Whole cell (upper panel) and magnified (lower panel) images are shown. Tau12-positive tau (red channel) represents full-length 2N4R tau. Both full-length and GFP-K18 tau were co-localized and formed fibular aggregates. Co-localization with p62 and tau aggregates (arrows) was partial. Bar for whole cell = 5 μm. Bar for magnified image = 1 μm; (**D**) F1B cell was immunostained with AT8 and p62 antibodies followed by formaldehyde fixation, and visualized by super-resolution microscopy. Whole cell (upper panel) and magnified (lower panel) images are shown. AT8 signal was not completely co-labeled with GFP, suggesting that phosphorylated tau was a part of tau fibrils. On the other hand, AT8-positive fibrils tended to co-localize with p62.

To examine the filamentous status of intracellular tau inclusions in stable cells, we performed fluorescence labeling with PBB5, which is one of the derivatives of tau PET ligand PBB3 [24]. PBB5 is a fluorescent β-sheet ligand similar to thioflavin S, and it selectively binds to filamentous tau aggregates. As a result, the tau inclusions in both D1C and F1B cells were positive with PBB5 fluorescence (Figure 3A,B), suggesting that the tau aggregates in the cells formed a β-sheet structure. To determine the structural characteristics of tau aggregates in cells, we dissected them in F1B cells by super-resolution structured illumination microscopy (SR-SIM) (Figure 3C,D). Tau12 immunoreactivity and GFP fluorescence were perfectly matched in the fibular-structured tau aggregates, suggesting that the cellular tau proteins generated filamentous aggregates containing both full-length tau and K18-tau (Figure 3C). These small tau filaments were mostly AT8 immunoreactive, but some portions of tau fibrils were not phosphorylated (Figure 3D, arrow), suggesting that tau phosphorylation is not essential for tau fibril formation.

### 2.4. p62 and Polyubiquitin Localization in Filamentous Tau Aggregates

**Figure 4:**
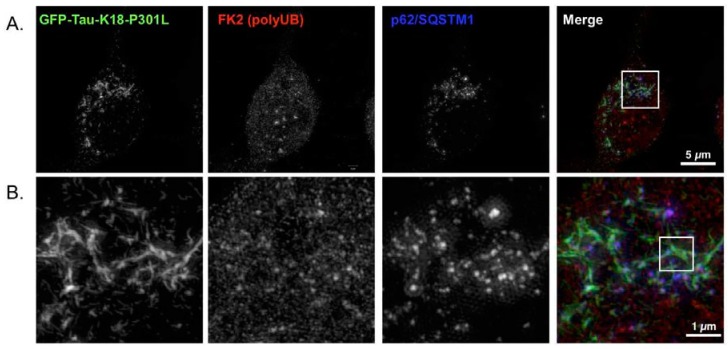

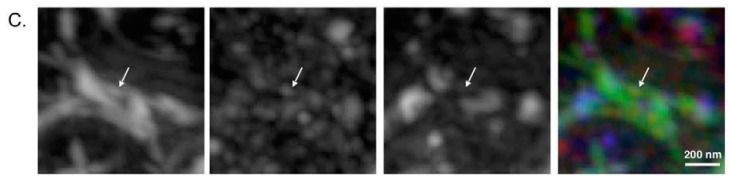
Visualization of polyubiquitinated tau by super-resolution microscopy. (**A**–**C**) Cell was immunostained with multiubiquitin (FK2) and p62 (p62c) antibodies following formaldehyde fixation, and was visualized by super-resolution microscopy (SR-SIM). Whole cell (**A**), magnified (**B**), and super-magnified (**C**) images are shown. Polyubiquitin signals were detected in p62 bodies, but only a little in tau fibrils (shown in **B**). FK2-positive patch (arrow in **C**) was observed in tau fibrils (merged image of **C**). Bar for super-magnified image = 200 nm.

To investigate whether these filamentous tau aggregates can be degraded by selective autophagy, we monitored the p62 localization by super-resolution microscopy. We found that both GFP-positive and Tau12-positive filamentous tau aggregates were partially co-localized with p62, but most of the tau filaments were p62-negative (Figure 3C,D). As p62 recognizes the polyubiquitin chains through its ubiquitin-association domain as substrates of selective autophagy [25,26,27], the small tau filaments may not be polyubiquitinated. To confirm this possibility, we monitored the ubiquitin distribution on tau filaments in F1B cells. As shown in Figure 4, multi-ubiquitin (FK2 antibody) signals were detected in p62 bodies, but they only weakly existed in tau fibrils (Figure 4A,B). The ubiquitination on tau fibrils was found as patch-like structures together with the fibrils (Figure 4C), suggesting that polyubiquitination against tau fibrils occurred after fibril formation, and protein degradation machinery may hardly recognize them as degradation substrates.

## 3. Discussion

In this study, we generated a novel cellular model of filamentous tau inclusions, in which the full-length human 2N4R tau isoform and GFP-fused repeat domain (K18) of tau with the P301L mutation were stably co-expressed and formed propagative aggregates. NFT-like features of tau inclusions in cells were confirmed by β-sheet ligand binding and super-resolution microscopy. Biochemical properties of the tau protein from tau fibril cell lines were compatible with those from aged rTg4510 mouse brains. Importantly, hyperphosphorylated tau defined by western blot analysis was recovered in both S1 (TBS-extractable) and P3 (sarkosyl-insoluble) fractions, which were aggregation intermediates and filamentous aggregates of pathological tau species, respectively. TBS-extractable hyperphosphorylated tau typically existed in the pathological status of tauopathy as an oligomer form of the tau protein [23]. Although the abundance of NFTs constituted with filamentous tau aggregates was significantly correlated with the severity of cognitive dysfunction in AD [28], accumulating evidence has indicated that NFTs themselves may not be neurotoxic [17,29]. Tau oligomers have attracted attention for the investigation of exact neurotoxic components of tau protein. Therefore, the present cellular model will be a powerful tool for the search for cytotoxic species of the tau protein.

Until the prion-like propagation model of tau aggregation was successfully developed [19], cellular models recapitulating basic features of the neurodegeneration of tauopathy were generated without robust tau aggregation. Since the intracellular accumulation of tau aggregates seems to be cytotoxic, destructive aggregates in cells with constitutively overexpressing tau protein may be eliminated during subcloning to establish stable transfectants. Our previous study showed that conditional transfectants expressing multiple tau isoforms (0N4R, 1N3R, and 1N4R) of wild-type human tau in M17D human neuroblastoma recapitulated the key characters of neurofibrillary degeneration (e.g., progressive tau aggregation, phosphorylation, truncation, tau fibril formation, and ubiquitination) [9]. Because tau expression was regulated by tetracycline or muristerone A treatment, the timing and the extent of tau overproduction were able to be controlled to generate filamentous tau inclusions in living cells [9]. Instead of using conditional expression systems, Diamond’s group first reported that extracellular tau aggregates induced filamentous tau aggregation in cells expressing the repeat domain (K18) of tau with both P301L and M337V mutations [10], and they also established several propagative tau K18 aggregate cell lines with different characters [19]. Lee V.M.Y. and her colleagues also developed a propagative tau aggregate cell line in which the GFP-fused full-length human tau with the P301L mutation was expressed under a conditional promoter, and they showed that tau inclusions were dynamic structures constantly undergoing fusion and fission without obvious cytotoxicity [11]. In contrast to their cell lines, our tau fibril cell lines express both a full-length and repeat domain of tau with the P301L mutation and contain tiny tau fibrils throughout the cytoplasm. The tau fibrils are composed of both variants of tau proteins and intertwine with each other to grow into large aggregates. Although these cell lines commonly have a consistent propagative property of tau aggregates from mother to daughter cells (Figure 1E), our cell lines exhibit cell death (Figure 1G) that is not reported in other cell lines. In our cells, tau inclusions grow over time, but their size is limited, indicating that the cells die before forming huge inclusions. This indicates that the tau inclusion has a cytotoxicity that gradually harms cells during their proliferation. On the other hand, our super-resolution microscopic analysis demonstrated phosphorylation-independent tau fibril formation in tau fibril cell lines (Figure 3D). To determine if the phosphorylation triggers tau aggregation, or if conformational change of the tau protein causes its hyperphosphorylation, further analysis of combinatory tau phosphorylations is required using the Phos-Tag SDS-PAGE methods [30]. Regardless, our cellular model will provide an attractive alternative for investigating molecular mechanisms of neurofibrillary degeneration in human tauopathy.

Our cell lines express both the human 2N4R tau isoform and K18 tau fragment with the P301L mutation. The cryo-EM study revealed that the core region of tau filaments from the AD brain was made of two identical protofilaments consisting of residues V306-F378 [31]. The N-terminal part of the cross-β structure is formed by the hexapeptide 306VQIVYK311 (PHF6), which is essential for tau aggregation [32,33]. Residues T373-F378 were observed to pack the interface of PHF6, which was absent in the K18 fragment. In our cell lines, filamentous tau aggregates contained both full-length and K18, suggesting that two types of protofilaments with distinct core structures existed in these tau aggregates. Presumably, filaments made of full-length 2N4R tau could represent human 4-repeat tauopathies, while K18 filaments might be different from PHFs or straight filaments observed in human tauopathies. It is of interest to note that the isolation of tau filaments in our tau fibril cell lines by cryo-EM seems the easiest way to identify core structures of K18 filaments.

p62 has been used as a marker to monitor autophagic activity. It has been reported that p62 co-localized with tau inclusions in human tauopathies [34,35], as well as in a mouse model expressing P301S mutant tau [36]. Our super-resolution microscopic analysis revealed that p62 was rarely co-localized with tau filaments in our cell lines. This suggests that the cellular protein degradation systems, including UPS and the p62-mediated aggrephagy pathway, cannot find the aggregated tau proteins to remove them through protein degradation processes. Therefore, great numbers of small tau fibrils can remain in cells and the elongated filaments can form large-sized tau inclusions. Although the cells with large tau inclusions will eventually die, small filamentous tau aggregates may not be toxic in our tau fibril cell lines, because these aggregates are stable in the cytosolic region and do not interfere with cellular division and proliferation (Figure 1F). Although the NFTs in human brain are ubiquitin-positive [37,38,39], it may be possible that the majority of tau fibrils are not polyubiquitinated but that the inclusions sequestrate a number of polyubiquitinated proteins, as well as p62. Further investigations are required to confirm these possibilities.

In conclusion, as far as we know, the present is a distinctive cellular model of tauopathy with features of NFT formation compatible with those observed in a tauopathy mouse model. The presented cell lines will be powerful instruments for investigating cellular mechanisms of toxic tau species, as well as for providing structural information of the tau filament core. Furthermore, our cellular model will provide cost-effective approaches to the development of high-throughput screening for potential therapeutics and formulate effective strategies for the treatment of tauopathies. 

## 4. Materials and Methods

### 4.1. Construction of Tau Fibril Cell Lines

Neuro2a cells were stably transfected with both hTau P301L 2N4R (pJTI-Tau-2N4R-P301L) and GFP-fused hTau P301L K18 (Q244-E372) fragments (GFP-Tau-K18-P301L). The N2a cell line continuously expressing both hTau P301L 2N4R tau and GFP-Tau-K18-P301L (clone 4C1) was generated as a non-aggregate control cell line. The recombinant Tau-K18-P301L fragment was purified from *E. coli* (Rosetta 2; Novagen, Madison, WI, USA) transformed by the pET54-Tau-K18-P301L plasmid according to methods described previously [19,20]. Briefly, His6-tagged Tau-K18-P301 fragments were induced with 1 mM IPTG for 3 h and cells were resuspended in 30 mM Tris-HCl pH8.0 and 500 mM NaCl, and then boiled at 98 °C for 10 min. After centrifugation (12,000 rpm, 10 min), the supernatant was passed through a PD-10 column for desalting. The desalted purified tau-K18 fragments were subjected to fibril formation by incubation with heparin (1/50 volume of 1000 U/mL; Novo Nordisk, Plainsboro, NJ) and 1 mM DTT (Sigma-Aldrich, St. Louis, MO, USA) at 37 °C for three days under shaking condition. Tau-K18 fibrils were collected by centrifugation and resuspended in sterilized PBS. Fibril formation was visually confirmed by Thioflavin S staining through a DAPI filter.

For the generation of tau aggregate cell lines (clone D1C and F1B), 4C1 cells were grown in 24-well plates and transfected with 2 µL of sonicated Tau-K13-P301L fibrils using Lipofectamine 3000. Transfected cells were re-plated onto 10-cm plates, colonies containing Tau aggregates were selected by fluorescence microscopy, and single colonies were re-plated to 10-cm plates again. Cells bearing Tau aggregates were sub-cloned twice, and then single cells were plated onto 96 well plates by limiting dilution. Wells containing a single cell were selected and the single cell-derived cell cultures bearing tau aggregates were named F1B and D1C, which were independently isolated from the first selection. It should be noted that these single cell-derived monoclonal cells with Tau aggregates spontaneously lose aggregates with certain probabilities, and cells with no aggregates appear in culture.

### 4.2. Immunofluorescence Microscopy

For co-localization studies, cells were grown on coverslips coated with poly-L-lysine (Sigma) in 24-well plates. Drug-treated N2a-derived cell lines were fixed in neutralized formaldehyde (Wako, Tokyo, Japan) or ice cold methanol-acetone (1:1) followed by neutralized formaldehyde fixation, blocked with 1% FBS and 0.1% Triton X-100 in PBS with 200 mM imidazole, 100 mM NaF, and a protease inhibitor cocktail (Sigma-Aldrich, St. Louis, MO, USA). Fixed cells were incubated with appropriate primary antibodies in blocking buffer, and then with AlexaFluor 568- or 647-conjugated anti- mouse (for Tau12, AT8, FK2) or guinea pig (for p62c) IgG (Life Technologies, Carlsbad, CA, USA) after washing with PBS + 0.1% Triton X-100, and were finally mounted in ProLong^®^ Diamond antifade mountant (Thermo Fisher Scientific, Waltham, MA, USA). Confocal microscopy was performed using a Zeiss LSM710 inverted confocal microscope equipped with a 100× oil lens with 2× zoom power. A whole-cell Z stack (each slice = 0.33 μm) was acquired, and maximum projection was created to visualize all fluorophores existing in a cell. Super-resolution structured illumination microscopy (SR-SIM) was performed using a Zeiss ELYRA super-resolution microscope equipped with a 100× oil lens (NA1.46) (Carl Zeiss Inc., Oberkochen, Germany). A whole-cell Z stack (each slice = 0.11 μm) was acquired with three rotations and analyzed for the reconstruction of super-resolution images. A maximum projection was created to visualize all fluorophores existing in a cell. All images were processed by Zen (Carl Zeiss, Oberkochen, Germany) and imageJ64 (NIH image, Bethesda, MD).

For PBB5 staining, methanol-fixed cells were incubated with 2 μM PBB5 (Styrl 7, Sigma-Aldrich, St. Louis, MO, USA) for 30 min at 25 °C. After cells were rinsed with 50% EtOH, both GFP and PBB5 fluorescence signals were captured with Keyence microscopy (BZ-X700, Keyence, Osaka, Japan).

For time-laps microscopy, cells were plated in 24 well-plates, and GFP and phase contrast images were automatically taken at several different regions at 10-min intervals for four days, using an incubator fluorescence microscope (Astec, Fukuoka, Japan) equipped with a 20x objective lens.

### 4.3. Mice

rTg4510 mice, tau responder mice, and tTA activator mice were obtained from the University of Florida. A parental mutant tau responder line in the FVB/N strain (Clea Inc. Tokyo, Japan) and a tTA activator line in the 129^+ter^/SV strain (Clea Inc., Tokyo, Japan) were generated and maintained, respectively. To make a tau responder line expressing the 4R0N isoform of human P301L mutant tau, cDNA was placed downstream of a tetracycline-operon-responder construct. The tTA activator system was placed downstream of the CaMKIIα promoter. Hemizygous mice from each parental line were crossed to produce F1 offspring containing rTg4510 mice [17]. For the present study, non-transgenic (non-tg) mice without any exogenous gene expression were used as control mice. All procedures involving mice were performed with approval of the National Institute of Radiological Sciences Institutional Animal Care and Use Committees (project # 07-1049-20, approved on 2 September 2015).

### 4.4. Tissue and Cell Lysate Extraction

For mouse brains, mice were euthanized by cervical dislocation in order to preserve the metabolic environment of the brain and to prevent artifacts that could alter the biochemical profiles of tau. Mouse brains were bisected down the midline to yield two hemispheres. The forebrain of the right hemisphere of each animal was snap frozen on dry ice and stored at −80 °C until processed, as previously described [18]. Tissues were homogenized in 10 volumes of Tris-buffered saline (TBS: 50 mM Tris/HCl [pH 7.4], 274 mM NaCl, 5 mM KCl, 1% protease inhibitor mixture (Sigma, St. Louis, MO, USA), 1% phosphatase inhibitor cocktail I & II (Sigma, St. Louis, MO, USA), 1 mM phenylmethylsulfonyl fluoride [PMSF]). For cell lysates, collected cells were homogenized in 300 μL of TBS (Figure 2A). The homogenates were centrifuged at 27,000× *g* for 20 min at 4 °C to obtain supernatant (S1) and pellet fractions. Pellets were further homogenized in five volumes for brains or 300 μL for cells (Figure 2A) of high salt/sucrose buffer (0.8 M NaCl, 10% sucrose, 10 mM Tris/HCl, [pH 7.4], 1 mM EGTA, 1 mM PMSF) and centrifuged as above. Supernatants were collected and incubated with sarkosyl (1% final concentration; Sigma, St. Louis, MO, USA) for 1 h at 37 °C, followed by centrifugation at 150,000× *g* for 1 h at 4 °C to obtain salt and sarkosyl-extractable (S3) and sarkosyl-insoluble (P3) fractions. The P3 pellet was re-suspended in TE buffer (10 mM Tris/HCl (pH 8.0), 1 mM EDTA) to a volume equivalent to half of that of the brain specimens used to produce brain homogenates or in 50 μL of TE buffer for cell lysates (Figure 2A).

### 4.5. Western Blotting

Fractionated tissue extracts were dissolved in SDS-sample buffer containing β-mercaptoethanol (2.5%). Heat-treated samples (55 °C for 15 min) were separated by gel electrophoresis on 10% Tris-glycine SDS-PAGE gels containing a 15-well comb (Invitrogen, Carlsbad, CA, USA) and transferred onto nitrocellulose membranes (BioRad Laboratories, Hercules, CA, USA). After blocking with 5% nonfat milk (dissolved in TBS with 0.1% Triton-X100), the membranes were incubated with various antibodies, washed to remove excess antibodies, and then incubated with peroxidase-conjugated goat anti-rabbit antibodies (1:5000; Jackson ImmunoResearch, West Grove, PA, USA) or anti-mouse IgG (1:5000; Jackson ImmunoResearch, USA). Bound antibodies were detected using an enhanced chemiluminescence system (ECL PLUS kit; PerkinElmer, Waltham, MA, USA). Western blot immunoreactivity was visualized by Amersham Imager 600 (GE Healthcare, Chicago, IL, USA). 

## References

[1] Lee V.M., Goedert M., Trojanowski J.Q. (2001). Neurodegenerative tauopathies. Annu. Rev. Neurosci..

[2] Iqbal K., Liu F., Gong C.X. (2016). Tau and neurodegenerative disease: The story so far. Nat. Rev. Neurol..

[3] Goedert M., Jakes R., Spillantini M.G., Hasegawa M., Smith M.J., Crowther R.A. (1996). Assembly of microtubule-associated protein tau into alzheimer-like filaments induced by sulphated glycosaminoglycans. Nature.

[4] Perez M., Valpuesta J.M., Medina M., Montejo de Garcini E., Avila J. (1996). Polymerization of tau into filaments in the presence of heparin: The minimal sequence required for tau-tau interaction. J. Neurochem..

[5] DeTure M., Ko L.W., Easson C., Yen S.H. (2002). Tau assembly in inducible transfectants expressing wild-type or ftdp-17 tau. Am. J. Pathol..

[6] Vogelsberg-Ragaglia V., Bruce J., Richter-Landsberg C., Zhang B., Hong M., Trojanowski J.Q., Lee V.M. (2000). Distinct ftdp-17 missense mutations in tau produce tau aggregates and other pathological phenotypes in transfected cho cells. Mol. Biol. Cell.

[7] Ebneth A., Godemann R., Stamer K., Illenberger S., Trinczek B., Mandelkow E. (1998). Overexpression of tau protein inhibits kinesin-dependent trafficking of vesicles, mitochondria, and endoplasmic reticulum: Implications for Alzheimer’s disease. J. Cell Biol..

[8] Dayanandan R., Van Slegtenhorst M., Mack T.G., Ko L., Yen S.H., Leroy K., Brion J.P., Anderton B.H., Hutton M., Lovestone S. (1999). Mutations in tau reduce its microtubule binding properties in intact cells and affect its phosphorylation. FEBS Lett..

[9] Ko L.W., Rush T., Sahara N., Kersh J.S., Easson C., Deture M., Lin W.L., Connor Y.D., Yen S.H. (2004). Assembly of filamentous tau aggregates in human neuronal cells. J. Alzheimer’s Dis..

[10] Frost B., Jacks R.L., Diamond M.I. (2009). Propagation of tau misfolding from the outside to the inside of a cell. J. Biol. Chem..

[11] Guo J.L., Buist A., Soares A., Callaerts K., Calafate S., Stevenaert F., Daniels J.P., Zoll B.E., Crowe A., Brunden K.R. (2016). The dynamics and turnover of tau aggregates in cultured cells: Insights into therapies for tauopathies. J. Biol. Chem..

[12] Varghese M., Santa-Maria I., Ho L., Ward L., Yemul S., Dubner L., Ksiezak-Reding H., Pasinetti G.M. (2016). Extracellular tau paired helical filaments differentially affect tau pathogenic mechanisms in mitotic and post-mitotic cells: Implications for mechanisms of tau propagation in the brain. J. Alzheimer's Dis..

[13] Woerman A.L., Aoyagi A., Patel S., Kazmi S.A., Lobach I., Grinberg L.T., McKee A.C., Seeley W.W., Olson S.H., Prusiner S.B. (2016). Tau prions from Alzheimer’s disease and chronic traumatic encephalopathy patients propagate in cultured cells. Proc. Natl. Acad. Sci. USA.

[14] Falcon B., Cavallini A., Angers R., Glover S., Murray T.K., Barnham L., Jackson S., O’Neill M.J., Isaacs A.M., Hutton M.L. (2015). Conformation determines the seeding potencies of native and recombinant tau aggregates. J. Biol. Chem..

[15] Kfoury N., Holmes B.B., Jiang H., Holtzman D.M., Diamond M.I. (2012). Trans-cellular propagation of tau aggregation by fibrillar species. J. Biol. Chem..

[16] Wu J.W., Hussaini S.A., Bastille I.M., Rodriguez G.A., Mrejeru A., Rilett K., Sanders D.W., Cook C., Fu H., Boonen R.A. (2016). Neuronal activity enhances tau propagation and tau pathology in vivo. Nat. Neurosci..

[17] Santacruz K., Lewis J., Spires T., Paulson J., Kotilinek L., Ingelsson M., Guimaraes A., DeTure M., Ramsden M., McGowan E. (2005). Tau suppression in a neurodegenerative mouse model improves memory function. Science.

[18] Sahara N., Deture M., Ren Y., Ebrahim A.S., Kang D., Knight J., Volbracht C., Pedersen J.T., Dickson D.W., Yen S.H. (2013). Characteristics of tbs-extractable hyperphosphorylated tau species: Aggregation intermediates in rtg4510 mouse brain. J. Alzheimer’s Dis..

[19] Sanders D.W., Kaufman S.K., DeVos S.L., Sharma A.M., Mirbaha H., Li A., Barker S.J., Foley A.C., Thorpe J.R., Serpell L.C. (2014). Distinct tau prion strains propagate in cells and mice and define different tauopathies. Neuron.

[20] Holmes B.B., Furman J.L., Mahan T.E., Yamasaki T.R., Mirbaha H., Eades W.C., Belaygorod L., Cairns N.J., Holtzman D.M., Diamond M.I. (2014). Proteopathic tau seeding predicts tauopathy in vivo. Proc. Natl. Acad. Sci. USA.

[21] Krammer C., Schatzl H.M., Vorberg I. (2009). Prion-like propagation of cytosolic protein aggregates: Insights from cell culture models. Prion.

[22] Greenberg S.G., Davies P. (1990). A preparation of alzheimer paired helical filaments that displays distinct tau proteins by polyacrylamide gel electrophoresis. Proc. Natl. Acad. Sci. USA.

[23] Ren Y., Sahara N. (2013). Characteristics of tau oligomers. Front. Neurol..

[24] Maruyama M., Shimada H., Suhara T., Shinotoh H., Ji B., Maeda J., Zhang M.R., Trojanowski J.Q., Lee V.M., Ono M. (2013). Imaging of tau pathology in a tauopathy mouse model and in alzheimer patients compared to normal controls. Neuron.

[25] Overbye A., Fengsrud M., Seglen P.O. (2007). Proteomic analysis of membrane-associated proteins from rat liver autophagosomes. Autophagy.

[26] Knaevelsrud H., Simonsen A. (2010). Fighting disease by selective autophagy of aggregate-prone proteins. FEBS Lett..

[27] Matsumoto G., Wada K., Okuno M., Kurosawa M., Nukina N. (2011). Serine 403 phosphorylation of p62/sqstm1 regulates selective autophagic clearance of ubiquitinated proteins. Mol. Cell.

[28] Grober E., Dickson D., Sliwinski M.J., Buschke H., Katz M., Crystal H., Lipton R.B. (1999). Memory and mental status correlates of modified braak staging. Neurobiol. Aging.

[29] Wittmann C.W., Wszolek M.F., Shulman J.M., Salvaterra P.M., Lewis J., Hutton M., Feany M.B. (2001). Tauopathy in drosophila: Neurodegeneration without neurofibrillary tangles. Science.

[30] Kimura T., Sharma G., Ishiguro K., Hisanaga S.I. (2018). Phospho-tau bar code: Analysis of phosphoisotypes of tau and its application to tauopathy. Front. Neurosci..

[31] Fitzpatrick A.W.P., Falcon B., He S., Murzin A.G., Murshudov G., Garringer H.J., Crowther R.A., Ghetti B., Goedert M., Scheres S.H.W. (2017). Cryo-em structures of tau filaments from Alzheimer’s disease. Nature.

[32] Von Bergen M., Friedhoff P., Biernat J., Heberle J., Mandelkow E.M., Mandelkow E. (2000). Assembly of tau protein into alzheimer paired helical filaments depends on a local sequence motif ((306)vqivyk(311)) forming beta structure. Proc. Natl. Acad. Sci. USA.

[33] Sahara N., Maeda S., Murayama M., Suzuki T., Dohmae N., Yen S.H., Takashima A. (2007). Assembly of two distinct dimers and higher-order oligomers from full-length tau. Eur. J. Neurosci..

[34] Kuusisto E., Salminen A., Alafuzoff I. (2001). Ubiquitin-binding protein p62 is present in neuronal and glial inclusions in human tauopathies and synucleinopathies. Neuroreport.

[35] Scott I.S., Lowe J.S. (2007). The ubiquitin-binding protein p62 identifies argyrophilic grain pathology with greater sensitivity than conventional silver stains. Acta Neuropathol..

[36] Schaeffer V., Lavenir I., Ozcelik S., Tolnay M., Winkler D.T., Goedert M. (2012). Stimulation of autophagy reduces neurodegeneration in a mouse model of human tauopathy. Brain J. Neurol..

[37] Mori H., Kondo J., Ihara Y. (1987). Ubiquitin is a component of paired helical filaments in Alzheimer’s disease. Science.

[38] Perry G., Friedman R., Shaw G., Chau V. (1987). Ubiquitin is detected in neurofibrillary tangles and senile plaque neurites of alzheimer disease brains. Proc. Natl. Acad. Sci. USA.

[39] Cripps D., Thomas S.N., Jeng Y., Yang F., Davies P., Yang A.J. (2006). Alzheimer disease-specific conformation of hyperphosphorylated paired helical filament-tau is polyubiquitinated through lys-48, lys-11, and lys-6 ubiquitin conjugation. J. Biol. Chem..

